# Rapid Serologic Test for Diagnosis of Yaws in Patients with Suspicious Skin Ulcers

**DOI:** 10.3201/eid2908.230608

**Published:** 2023-08

**Authors:** Clara Suñer, Lucy N. John, Wendy Houinei, Maria Ubals, Dan Ouchi, Andrea Alemany, Cristina Galván-Casas, Michael Marks, Oriol Mitjà, Martí Vall-Mayans, Camila G. Beiras

**Affiliations:** Fight Infections Foundation, Badalona, Spain (C. Suñer, L.N. John, M. Ubals, D. Ouchi, A. Alemany, C. Galván-Casas, O. Mitjà, M. Vall-Mayans, C.G. Beiras);; University Hospital Germans Trias i Pujol, Badalona (C. Suñer, M. Ubals, A. Alemany, O. Mitjà, M. Vall-Mayans, C.G. Beiras); ﻿; National Department of Health, Port Moresby, Papua New Guinea (L.N. John, W. Houinei);; University of Papua New Guinea, Port Moresby (L.N. John, O. Mitjà);; Hospital Universitario de Móstoles, Madrid, Spain (C. Galván-Casas);; London School of Hygiene & Tropical Medicine, London, UK (M. Marks);; Hospital for Tropical Diseases, London (M. Marks); University College London, London (M. Marks)

**Keywords:** yaws, Treponema pallidum subsp. pertenue, rapid diagnostic test, Dual Path Platform, DPP, skin ulcer, neglected diseases, bacteria, Papua New Guinea

## Abstract

﻿The Chembio DPP (Dual Path Platform) Syphilis Screen & Confirm kit (https://chembio.com) is a rapid serologic test that can be used to diagnose yaws. We evaluated its capacity to detect patients with ulcers that tested PCR positive for *Treponema pallidum* subsp. *pertenue*. DPP detected 84% of ulcers that were positive by PCR.

Yaws is a neglected tropical disease caused by *Treponema pallidum* subsp. *pertenue* (TPE) that causes cutaneous ulcers. It predominantly affects children living in remote communities. The World Health Organization designated 2020 as the year that yaws would be eradicated. That year, 87,877 clinically suspected cases were reported, but only 346 (from 7 countries, primarily western Pacific countries) were confirmed as yaws (*1*). Thus, confirming a yaws diagnosis on the basis of ulcerative lesions remains challenging for yaws eradication (*2*). Standard tests for yaws diagnosis require sample processing in a laboratory, which is often unavailable in rural health centers where yaws is endemic (*3*). A mainstay for achieving yaws eradication is integration of point-of-care tests into surveillance strategies.

The Chembio DPP (Dual Path Platform) Syphilis Screen & Confirm kit (https://chembio.com) has been proposed as a point-of-care test for confirming yaws as the cause of tropical ulcers. This lateral-flow immune-chromatographic rapid test simultaneously detects antibodies against *T. pallidum* (T line) and non–*T. pallidum* (NT line) antigens in blood (*4*,*5*). Aside from the qualitative result, which is readable with the naked eye, a quantitative measurement that uses optical density microreaders has been developed. According to previous reports, the qualitative reading of the DPP NT line has 80% sensitivity and 96% specificity for TPE compared with the rapid plasma reagin serologic test (*6*). However, the ability of the DPP test to identify active TPE in skin ulcers with a positive PCR result has not been established.

We assessed the ability of qualitative and quantitative measurements of DPP to identify active TPE in tropical ulcers. We used data from a community trial of patients with skin ulcers suggestive of yaws, conducted in Namatanai, Papua New Guinea, during 2018–2019 (*7*). We compared ulcer PCR results for TPE with serologic results of the DPP test T line, NT line, or both, read by the naked eye or by using the quantitative reader. The study protocol was approved by the Medical Research Advisory Committee of the Papua New Guinea National Department of Health. Participants provided written informed consent for collection of biological samples.

We tested samples from 995 suspicious skin ulcers by using DPP and PCR. The mean age of participants was 15.9 (SD ±14.1) years, and 454 (46.5%) were female. Median ulcer duration was 4 (interquartile range 2–8) weeks; median size was 2.0 (interquartile range 1.5–2.5) cm. For 745 (78.1%) case-patients, the ulcer was a first episode, and 662 (72.4%) had only 1 ulcer at the time of examination. Overall, 287 (28.8%) had a positive TPE PCR result. Ulcers positive by PCR were more frequently found in younger persons with only 1 ulcer, which was long-lasting and a first episode (Appendix Table 1). DPP reader results were available for 828 (83.2%) of the ulcers, of which PCR results were positive for 247 (29.8%).

Sensitivity of DPP detection of TPE PCR-positive cases with the naked eye was highest when we used the NT line, and specificity was highest when we used a combination of T and NT lines (Table). Using the values from the reader, we evaluated the optimal combination of cutoff values for the DPP T and NT lines, which maximized the sum of sensitivity and specificity to distinguish lesions that were positive and negative for TPE by PCR (Figure). That combination (T ≥1 and NT ≥28) provided sensitivity of 75.7% and specificity of 77.6% (Table). The subanalysis of DPP performance according to participants’ characteristics showed higher specificity for children <7 years of age and for adults (>18 years of age) (Appendix Tables 2, 3).

**Table:** Performance of DPP Syphilis Screen & Confirm kit in study of rapid serologic test for diagnosis of yaws in patients with suspicious skin ulcers

Detection technique	PCR negative		Specificity, % (95% CI)	PCR positive		Sensitivity, % (95% CI)	Sensitivity + specificity, %	PPV, % (95% CI)	NPV, % (95% CI)
	Detected, no.	Not detected, no.		Detected, no.	Not detected, no.				
Naked eye									
T line	357	351	49.6 (45.8–53.3)	227	60	79.1 (73.9–83.7)	128.7	38.9 (34.9–43.0)	85.4 (81.6–88.7)
NT line	278	430	60.7 (57.0–64.4)	241	46	84.0 (79.2–88.0)	144.7	46.4 (42.1–50.8)	90.3 (87.3–92.8)
T and NT line	222	486	68.6 (65.1–72.1)	214	73	74.6 (69.1–79.5)	143.2	49.1 (44.3–53.9)	86.9 (83.9–89.6)
Reader									
T >1 and NT reader >28†	130	451	77.6 (74.0–81.0)	187	60	75.7 (69.9–80.9)	153.3	59.0 (53.4–64.5)	88.3 (85.2–90.9)

*DPP (Dual Path Platform) Syphilis Screen & Confirm kit, Chembio (https://chembio.com). NPV, negative predictive value; NT line, antibodies against non–*Treponema pallidum* antigen; PPV, positive predictive value; T line, antibodies against *T. pallidum* antigen. †Values used as cutoff with the DPP reader were calculated to maximize the sum of sensitivity and specificity (Figure).

**Figure:**
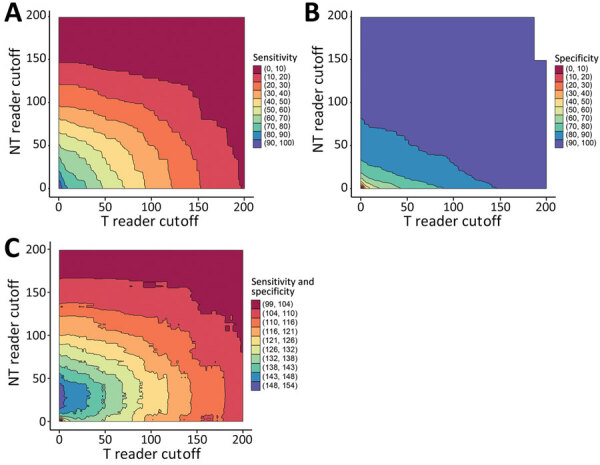
Performance of DPP Syphilis Screen & Confirm kit (Chembio, https://chembio.com) as rapid serologic test for diagnosis of yaws, with combinations of cutoff values for the T and NT line, measured with DPP reader. The heatmap legend indicates the range of sensitivity (A), specificity (B), or the sum of sensitivity and specificity (C) that each combination of cutoff values would provide. The sensitivity and specificity obtained by using different reader cutoff values for the T and NT lines separately are shown in the Appendix Figure. DPP, Dual Path Platform; NT, antibodies against non–*Treponema pallidum* antigen; T, antibodies against *T. pallidum* antigen.

The proportion of cutaneous ulcers in yaws-endemic regions that were TPE positive declined from 44% to 8% after 1 round of azithromycin mass drug administration (delivery to all consenting members of target community, regardless of diagnosis) (*7**,**8*). In that context, the combined T and NT lines, recommended for surveillance, would be sufficient to partially detect ongoing yaws transmission, but PCR confirmation would be required to discern TPE ulcers from latent cases or false-positive results (Appendix Table 4).

The DPP test can provide up to 84% sensitivity for detecting TPE PCR-positive ulcers with the naked eye when using the NT line, although the specificity of this strategy is low (61%). The automatic reader did not increase sensitivity. Our results should be interpreted by bearing in mind that the reference and index tests provide information regarding different features or manifestations of yaws: skin ulcers with TPE DNA and serologic activity of the host. Therefore, different disease phases such as incubation period or latency, or other confounders such as syphilis infections, may contribute to conflicting PCR and DPP results.

Overall, the DPP test showed a reasonably high capacity to identify yaws in persons with TPE PCR-confirmed ulcers. That level of performance is suitable for qualitatively identifying ongoing transmission of yaws in the community during the late phases of eradication. However, for individual diagnoses, PCR confirmation of suspicious ulcers remains necessary; new point-of-care tests with higher sensitivity and specificity would be valuable.

Appendix Additional results for study of rapid serologic test for diagnosis of yaws in patients with suspicious skin ulcers.
