# Supplementary material for: Rapid Serologic Test for Diagnosis of Yaws in Patients with Suspicious Skin Ulcers
Source: Emerg Infect Dis. 2023 Aug;29(8):1682–4. doi: 10.3201/eid2908.230608 (PMC10370868; doi:10.3201/eid2908.230608)
Supplement: Appendix — Additional results for study of rapid serologic test for diagnosis of yaws in patients with suspicious skin ulcers. [file 23-0608-Techapp-s1.pdf]

Article DOI: https://doi.org/10.3201/eid2908.230608

EID cannot ensure accessibility for supplementary materials supplied by authors. Readers who have difficulty accessing supplementary content should contact the authors for assistance.

Rapid Serologic Test for Diagnosis of Yaws in Patients with Suspicious Skin Ulcers

Appendix

Appendix Table 1. Characteristics of patients with TPE-positive and TPE-negative PCRs

		PCR result			
Characteristic	Total	Negative	Positive	P-value	% Missing
N	995	708	287		
Age (mean (SD))	15.86 (14.1)	17.79 (15.2)	11.08 (9.3)	<0.001	2.5
≤7y	252 (26.0)	146 (21.1)	106 (38.0)	<0.001	0
8-12y	250 (25.8)	159 (23.0)	91 (32.6)		
13-18y	245 (25.3)	194 (28.1)	51 (18.3)		
>18y	223 (23.0)	192 (27.8)	31 (11.1)		
Gender, N (%)					
Male	522 (53.5)	361 (51.9)	161 (57.3)	0.148	1.9
Female	454 (46.5)	334 (48.1)	120 (42.7)		
Ulcer duration, in weeks (median [IQR])	4.00 [2.00, 8.00]	3.50 [2.00, 6.00]	4.00 [2.00, 8.00]	0.001	9.6
≤4 wks	561 (62.4)	405 (64.1)	156 (58.4)	0.127	0
>4 wks	338 (37.6)	227 (35.9)	111 (41.6)		
Ulcer size, in cm (median [IQR])	2.00 [1.50, 2.50]	2.00 [1.50, 3.00]	2.00 [1.50, 2.50]	0.853	11.5
≤2cm	592 (67.2)	411 (66.2)	181 (69.6)	0.362	0
>2cm	289 (32.8)	210 (33.8)	79 (30.4)		
First episode, N (%)					
No	209 (21.9)	167 (24.6)	42 (15.3)	0.002	4.1
Yes	745 (78.1)	512 (75.4)	233 (84.7)		
Number of ulcers, N (%)					
1	662 (72.4)	457 (70.2)	205 (77.9)	0.021	8.1
2	147 (16.1)	106 (16.3)	41 (15.6)		
3	46 (5.0)	39 (6.0)	7 (2.7)		
>3	59 (6.5)	49 (7.5)	10 (3.8)		

Appendix Table 2. Performance of DPP according to the characteristics of individuals and ulcers, by reader ≥1 and NT ≥28

		DPP negative			DPP positive (Reader T≥ 1 and NT≥ 28)			
Characteristic	Category	PCR-detected	PCR- not detected	Specificity (95% CI)	PCR detected	PCR not detected	Sensitivity (95% CI)	Sum Specificity and Sensitivity
Age	≤7y	15	109	87.9% (80.8-93.1)	68	18	79.1% (69.0-87.1)	167.0%
	8-12y	49	80	62.0% (53.1-70.4)	60	22	73.2% (62.2-82.4)	135.2%
	13-18y	44	113	72.0% (64.3-78.8)	32	12	72.7% (57.2-85.0)	144.7%
	>18y	18	141	88.7% (82.7-93.2)	23	5	82.1% (63.1-93.9)	170.8%
Gender	Male	72	232	76.3% (71.1-81.0)	103	32	76.3% (68.2-83.2)	152.6%
	Female	58	209	78.3% (72.8-83.1)	81	26	75.7% (66.5-83.5)	154.0%
First episode	No	30	96	76.2% (67.8-83.3)	22	8	73.3% (54.1-87.7)	149.5%
	Yes	93	336	78.3% (74.1-82.1)	158	51	75.6% (69.2-81.3)	153.9%

Characteristic	Category	DPP negative PCR-detected	DPP negative PCR- not detected	Specificity (95% CI)	DPP positive (Reader T≥ 1 and NT≥ 28) PCR detected	DPP positive (Reader T≥ 1 and NT≥ 28) PCR not detected	Sensitivity (95% CI)	Sum Specificity and Sensitivity
Ulcer Duration	≤4 weeks	67	272	80.2% (75.6-84.3)	105	31	77.2% (69.2-84.0)	157.4%
	>4 weeks	53	133	71.5% (64.4-77.9)	71	25	74.0% (64.0-82.4)	145.5%
Ulcer Number	1	93	277	74.9% (70.1-79.2)	129	46	73.7% (66.5-80.1)	148.6%
	2	12	77	86.5% (77.6-92.8)	29	7	80.6% (64.0-91.8)	167.1%
	3	7	29	80.6% (64.0-91.8)	6	0	100% (54.1-100)	180.6%
	>3	7	33	82.5% (67.2-92.7)	8	1	88.9% (51.8-99.7)	171.4%
Ulcer size	≤2cm	77	257	77.0% (72.1-81.4)	114	41	73.6% (65.9-80.3)	150.5%
	>2cm	39	136	77.7% (70.8-83.7)	53	14	79.1% (67.4-88.1)	156.8%

Appendix Table 3. Performance of DPP according to the characteristics of individuals and ulcers, by T and NT, naked eye

Characteristic	Category	DPP negative PCR-detected	DPP negative PCR- not detected	Specificity (95% CI)	DPP positive (T and NT, naked eye) PCR detected	DPP positive (T and NT, naked eye) PCR not detected	Sensitivity (95% CI)	Sum Specificity and Sensitivity
Age	≤7y	32	114	78.1% (70.5-84.5)	76	30	71.7% (62.1-80.0)	149.8%
	8-12y	68	91	57.2% (49.2-65.0)	68	23	74.7% (64.5-83.3)	132.0%
	13-18y	72	122	62.9% (55.7-69.7)	39	12	76.5% (62.5-87.2)	139.4%
	>18y	46	146	76.0% (69.4-81.9)	25	6	80.7% (62.5-92.6)	156.7%
Gender	Male	125	236	65.4% (60.2-70.3)	122	39	75.8% (68.4-82.2)	141.2%
	Female	92	242	72.5% (67.3-77.2)	89	31	74.2% (65.4-81.7)	146.6%
First episode	No	65	102	61.1% (53.2-68.5)	28	14	66.7% (50.5-80.4)	127.7%
	Yes	145	367	71.7% (67.6-75.6)	177	56	76.0% (70.0-81.3)	147.7%
Ulcer Duration	≤4 weeks	99	306	75.6% (71.1-79.7)	114	42	73.1% (65.4-79.9)	148.6%
	>4 weeks	96	131	57.7% (51.0-64.2)	89	22	80.2% (71.5-87.1)	137.9%
Ulcer Number	1	151	306	67.0% (62.4-71.3)	153	52	74.6% (68.1-80.4)	141.6%
	2	23	83	78.3% (69.2-85.7)	31	10	75.6% (59.7-87.6)	153.9%
	3	10	29	74.4% (57.9-87.0)	7	0	100.0% (59.0-100.0)	174.4%
	>3	16	33	67.4% (52.5-80.1)	9	1	90.0% (55.5-99.8)	157.4%
Ulcer size	≤2cm	127	284	69.1% (64.4-73.5)	135	46	74.6% (67.6-80.8)	143.7%
	>2cm	66	144	68.6% (61.8-74.8)	63	16	79.8% (69.2-88.0)	148.3%

Appendix Table 4. Estimated DPP and PCR results for 1,000 ulcers tested, according to different prevalence values in the field

Yaws prevalence among ulcers	Type of test (naked eye)	Specificity (95% CI)	Sensitivity (95% CI)	DPP - PCR + cases (95% CI)	DPP + PCR - Cases† (95% CI)	DPP - PCR - cases (95% CI)	DPP + PCR + cases (95% CI)
1%	T and NT positives	68.6% (65.1-72.1)	74.6% (69.1-79.5)	3 (2-3)	311 (276-346)	679 (644-714)	7 (7-8)
8%	T and NT positives	68.6% (65.1-72.1)	74.6% (69.1-79.5)	20 (16-25)	289 (257-321)	631 (599-663)	60 (55-64)
44%	T and NT positives	68.6% (65.1-72.1)	74.6% (69.1-79.5)	112 (90-136)	176 (156-195)	384 (365-404)	328 (304-350)
1%	T line positive	49.6% (45.8-53.3)	79.1% (73.9-83.7)	2 (2-3)	499 (462-537)	491 (453-528)	8 (7-8)
8%	T line positive	49.6% (45.8-53.3)	79.1% (73.9-83.7)	17 (13-21)	464 (430-499)	456 (421-490)	63 (59-67)
44%	T line positive	49.6% (45.8-53.3)	79.1% (73.9-83.7)	92 (72-115)	282 (262-304)	278 (256-298)	348 (325-368)
1%	NT line positive	60.7% (57.0-64.4)	84.0% (79.2-88.0)	2 (1-2)	389 (352-426)	601 (564-638)	8 (8-9)
8%	NT line positive	60.7% (57.0-64.4)	84.0% (79.2-88.0)	13 (10-17)	362 (328-396)	558 (524-592)	67 (63-70)
44%	NT line positive	60.7% (57.0-64.4)	84.0% (79.2-88.0)	70 (53-92)	220 (199-241)	340 (319-361)	370 (348-387)

*Yaws prevalence among cutaneous ulcers in an endemic region declines from 44% to 8% after one round of azithromycin mass drug administration (7). 1% prevalence is expected in the late phase of eradication.
†These cases might be DPP false positive results, or latent yaws cases that are DPP-positive despite not having yaws-positive ulcers, among others.

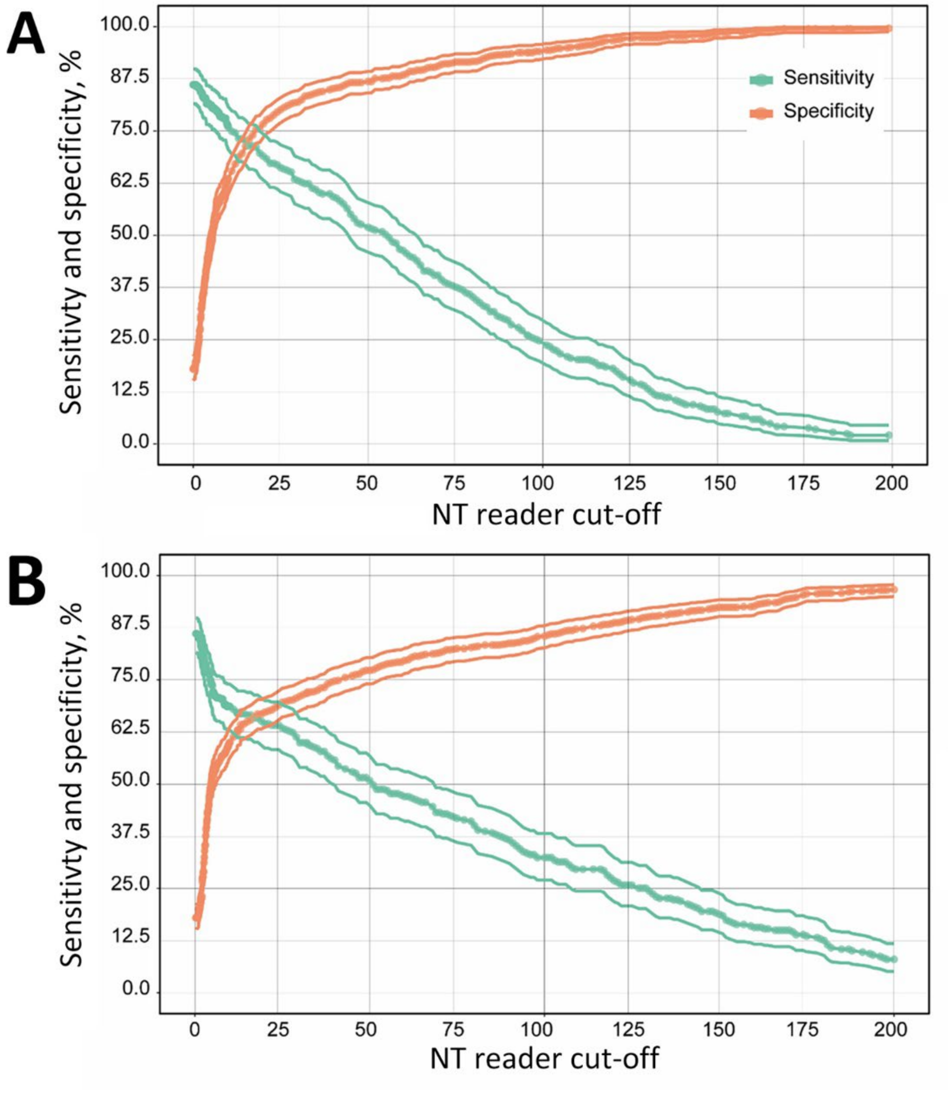

Appendix Figure. Sensitivity and specificity of the DPP using the reader at different cutoff values for the NT-line and the T-line separately.
